# Evidence for complex contagion models of social contagion from observational data

**DOI:** 10.1371/journal.pone.0180802

**Published:** 2017-07-07

**Authors:** Daniel A. Sprague, Thomas House

**Affiliations:** 1 Spectra Analytics, 40-42 Scrutton St, London EC2A 4PP, United Kingdom; 2 School of Mathematics, University of Manchester, Manchester, M13 9PL, United Kingdom; Universitat Rovira i Virgili, SPAIN

## Abstract

Social influence can lead to behavioural ‘fads’ that are briefly popular and quickly die out. Various models have been proposed for these phenomena, but empirical evidence of their accuracy as real-world predictive tools has so far been absent. Here we find that a ‘complex contagion’ model accurately describes the spread of behaviours driven by online sharing. We found that standard, ‘simple’, contagion often fails to capture both the rapid spread and the long tails of popularity seen in real fads, where our complex contagion model succeeds. Complex contagion also has predictive power: it successfully predicted the peak time and duration of the ALS Icebucket Challenge. The fast spread and longer duration of fads driven by complex contagion has important implications for activities such as publicity campaigns and charity drives.

## Introduction

### Social influence

There is a large body of evidence—which is increasingly quantitative—that the effect of social influence can be a significant driver of human behaviour. Improved understanding of this phenomenon should help to predict various phenomena of interest, for example how well public-health interventions will work, or the use of ‘nudges’ in public policy [1–7].

In particular, the work of Christakis and Fowler [7] analysed longitudinal social network and health data from the Framingham Heart Study and showed that if an individual had a friend, sibling, or spouse who had become obese in a given time interval then that individual was significantly more likely to also become obese. Similar results were also found when studying the cessation of smoking [6]. This proved controversial; it has been shown that social influence cannot be distinguished from homophily, or the clustering of individuals who are similar, in observational studies [8]. Aral et al. [9] try to determine an upper bound for the importance of social influence for behaviour spread, and find that for the adoption of a particular social media app at least half of the observed adoption events can be attributed to homophily. This discussion highlights the difficulty of using observational data to distinguish the effect of individual-level factors, in the form of homophily, from social influence. This same difficulty is not present in experimental data, however. Bond et al. performed a randomised controlled trial over Facebook to find evidence for social influence on the decision to vote [3]. By sending direct messages to ‘seed’ nodes in a network, and then tracking the behaviour of their contacts, the experimenters showed that individuals were significantly more likely to vote if one of their close friends had received a message. In a study also related to electronically mediated real-world behaviour, Centola [5] placed individuals in an artificially-structured online community in which users were informed about the health activities of their assigned contacts. This experiment showed that social signals significantly increased the likelihood of an individual taking part in a behaviour, and that up to three additional social signals significantly increased this likelihood even further. Taken together, these studies show that while individual-level factors are significant, social influence is also important in determining health behaviours.

### Previous models

Models of social influence have taken three main forms: experimental generalisations, agent-based models, and compartmental models. Experimental generalisations take historical data on the spread of a behaviour and try to find functional forms which match that data. One of the first examples of this approach was by Bass [10], who created a model of product adoption based on the idea of innovators and imitators. More recent attempts include fitting a variety of statistical distributions to the popularity of Internet memes [11]. The main disadvantage to this approach is that it does not provide a mechanistic model for social influence, and hence does not provide much insight into individual-level processes.

Agent-based models take almost the opposite approach to the experimental generalisations mentioned above, in that they simulate all of the individual- (or ‘agent’-) level processes occurring and then try to calibrate the model by matching the aggregate behaviour to data [12, 13]. Agent-based models are useful tools for reproducing the complex phenomena observed in real systems, but it is extremely difficult to fit their parameters to data well.

Compartmental models put each individual in the population into one of a certain number of states, or compartments. Only the number of individuals in each compartment and the transitions between them are tracked, and hence the number of dimensions of the system can be much less than an equivalent agent-based model. This in turn allows a compartmental model to be fitted to data more easily than agent-based models, while remaining a mechanistic description of the underlying system. Treating social influence in this compartmental way has a long history, an example being Dietz [14] who developed a model for the spreading of rumours similar to models from epidemiology. In fact, much of the social influence literature using compartmental models has been based on the SIRS model of an epidemic. In the SIRS model there are three compartments: susceptible (S), infectious (I), and recovered (R). Susceptible individuals have not yet been infected with the disease, infected individuals currently have the disease and are spreading it, and recovered individuals have had the disease but are no longer spreading it. In the standard SIRS model used to model infections [15], individuals moving between these compartments are modelled by a continuous time Markov chain with events and rates

$$\begin{array}{c} \begin{array}{ccccc} \left(S,I\right) & \to & \left(S-1,I+1\right) & & \;\text{at}\;\text{rate}\;\beta SI\text{,}\\ \left(S,I\right) & \to & \left(S,I-1\right) & & \;\text{at}\;\text{rate}\;\gamma I\text{,}\\ \left(S,I\right) & \to & \left(S+1,I\right) & & \;\text{at}\;\text{rate}\;\delta R\text{.} \end{array} \end{array}$$ (1)

This standard model can be modified by changing the functions for the rates, and by adding or removing compartments. For models of social influence on behaviour, the ‘infectious’ compartment represents individuals taking part in a behaviour and spreading it, and ‘recovered’ means the individual is no longer influencing others to take part in the behaviour. Many previous studies of social influence modify the standard model by changing the rates at which at which individuals move between compartments. Isham et al. [16], for example, developed a model for rumours on a network based on the SIR model modified to include ‘stiflers’ who cause infectious individuals to recover at a faster rate. One important additional source of realism is to consider the impact of contact network structure on spreading dynamics, however if the degree distribution of the network is not too heterogeneous and other properties such as clustering, assortativity and path length are not too far from a random graph then dynamics such as Eq (1) should be a good approximation [17].

Very few compartmental models for social influence modify the form of the infection term in the standard model. However, as shown in experimental studies [5], there is significant evidence that the form of ‘infection’ in social influence is different to that in a biological epidemic. The important difference is the number of exposures to infection that an individual must receive before becoming infected: in biological infection only one source of infection is required for a non-zero probability of infection, whereas in social influence multiple sources are required. Dodds and Watts [18], for example, generalise the SIS model to allow for infection processes that require multiple exposures.

### Testing complex contagion

While the work of Centola involved a controlled study to test for effects of complex contagion, if this is a strong effect in general then it should be possible to find evidence for it in observational data at the population level. In this paper, we set up simple and complex contagion models for populations, which we compare to search-interest data on photo fads—i.e. electronically mediated real-world behaviours—using maximum likelihood estimation and information theoretic model selection. We show using these methods that complex contagion is strongly favoured as a model of social influence, which can then be used predictively.

## Materials and methods

### Mathematical definition of the model

We propose here a general modelling framework based on a non-linear continuous-time stochastic process that enables us to capture most existing models of behavioural contagion as special cases. We start with a vector of non-independent integer random variables, **X**(*t*) = (*S*, *Y*_1_, …, *Y*_*n*_, *R*), where *S* represents the number of individuals not engaging in the behaviour who might start if exposed to it; *R* represents the number of individuals not engaging in the behaviour who will not start if exposed, and *Y*_*i*_ represents the number of individuals engaging in the behaviour of ‘type’ *i*. The events and transition rates defining this stochastic process are given by

$$\begin{array}{c} \begin{array}{ccccc} \left(S,Y_{1},\ldots ,R\right) & \to & \left(S-1,Y_{1}+1,\ldots ,R\right) & & \text{at}\;\text{rate}\;Sf(S,Y_{1},\ldots ,R)\text{,}\\ \left(\ldots ,Y_{i},Y_{i+1},\ldots \right) & \to & \left(\ldots ,Y_{i}-1,Y_{i+1}+1,\ldots \right) & & \text{at}\;\text{rate}\;Y_{i}g_{i}\left(S,Y_{1},\ldots ,R\right)\text{,}\\ \left(S,Y_{1},\ldots ,R\right) & \to & \left(S+1,Y_{1},\ldots ,R\right) & & \text{at}\;\text{rate}\;Rh(S,Y_{1},\ldots ,R)\text{.} \end{array} \end{array}$$ (2)

This model is ‘SIRS-like’, but if *h* → ∞ it becomes ‘SIS-like’, and if *h* → 0 it becomes ‘SIR-like’. The general model can also be specialised to fit many spreading situations. We will now outline the specific choices that we have made to formulate models for the spread of photo fads.

#### Complex contagion model

We follow the broad mathematical approach of [19] that seeks to capture the effects of ‘complex contagion’ seen in the work of Centola [5, 20, 21] in a relatively simple functional form. In the basic form of this model, each individual canvasses *C* contacts selected from the rest of the population uniformly at random, and if the number of these contacts taking part in a behaviour is greater than some threshold *τ* then the individual changes state.

In terms of the ‘infectious’ classes that spread behaviour, we use two: *Y*_1_ = *I* for those new to the fad and *Y*_2_ = *J* for others participating in the fad. This represents the greater attention given to novel behaviour, and from a technical point of view stops the fad-free fixed point of the system from being stable as would be the case in simpler models [19]. Since the transition between these two states is just a question of time spent spreading behaviour, we simply assume individuals moving from *I* to *J* at a constant rate *ϵ*; this parameter affects the duration of the trend with high values of *ϵ* leading to sharper peaks and low values lead to wider ones. For the other transitions we use complex contagions giving the following rate functions:

$$\begin{array}{c} \begin{array}{ccc} f(I,J) & = & \beta \sum_{k=\tau_{i}}^{C}\sum_{l=0}^{C}\;\text{Multi}\;(k,l|I/N,J/N,C)\\ & & +\beta \sum_{k=0}^{\tau_{i}-1}\sum_{l=\tau_{j}}^{C}\;\text{Multi}\;(k,l|I/N,J/N,C)\text{;}\\ g_{1} & = & \epsilon \text{;}\\ g_{2}\left(J\right) & = & \sum_{y=\tau_{r}}^{C}\;\text{Bin}\left(y|J/N,C\right)\text{,}\\ h & = & 0\text{.} \end{array} \end{array}$$ (3)

We have, therefore, assumed that individuals do not return to a fad in which they have previously participated. We note that there are various other well-motivated modelling choices that could be made at this stage, and that while a systematic comparison of such approaches is beyond the scope of the current work we believe it would be an interesting direction for future study.

If we consider a large fixed population of size *N* = *S* + *I* + *J* + *R* then the stochastic Model (2) with choices Eq (3) as above can be approximated by the following system of ODEs [22, 23], with error *O*(*N*^−1/2^):

$$\begin{array}{c} \frac{dS}{dt}=-f\left(I,J\right)S\text{,}\;\frac{dI}{dt}=f\left(I,J\right)S-\epsilon \text{,}\;\frac{dJ}{dt}=\epsilon I-g_{2}\left(J\right)J\text{.} \end{array}$$ (4)

What distinguishes this ODE system from many other approaches to social contagion is the presence of high-order polynomials on the right-hand side of the equations. Roughly speaking, this model is similar to some ‘excitable’ models in mathematical biology which exhibit fast growth and shrinkage [24, 25], and this turns out to be the aspect of complex contagion that causes it to be preferred over simple contagion.

#### Simple contagion model

Our simple contagion model is a straightforward modification of the standard SIR model:

$$\begin{array}{c} \frac{dS}{dt}=-\left(\beta_{i}I+\beta_{j}J\right)S\text{,}\;\frac{dI}{dt}=\left(\beta_{i}I+\beta_{j}J\right)S-\epsilon \text{,}\;\frac{dJ}{dt}=\epsilon I-\gamma J\text{.} \end{array}$$ (5)

Our aim will be to fit Eqs (4) and (5) to data to look for population-level evidence that can discriminate between simple and complex contagion. For both models, we will also need to fit an initial number *I*(0) participating in the fad; we will also assume that *J*(0) = *R*(0) = 0 and so the rest of the population is initially in the *S* compartment so that *S*(0) = *N* − *I*(0).

We can also now make our verbal argument above about ‘excitable’ models more quantitatively. Consider the special case of our models in which *C* = *τ*_*i*_ = 2 and *ϵ* = 0. Early in the epidemic, for the simple contagion model, making the special choices *β*_*i*_ = 1/*N* and *I*(0) = 1 for simplicity, we will be able to make the large-*N* approximation

$$\begin{array}{c} \frac{dI}{dt}\approx I\;\Rightarrow \;I\left(t\right)\approx e^{t}\text{,} \end{array}$$ (6)

i.e. exponential early growth. For the complex contagion model, making the special choices *β* = *N* and *I*(0) = 1 for simplicity, we will have the large-*N* approximation

$$\begin{array}{c} \frac{dI}{dt}\approx I^{2}\;\Rightarrow \;I\left(t\right)\approx \frac{1}{1-t}\text{,} \end{array}$$ (7)

which represents super-exponential early growth. In both the simple and complex models *I*(*t*) will eventually stop growing due to non-linear effects as *S*(*t*) decreases, but the early growth of the complex model will be much more ‘explosive’, which is a feature that we will see in real data.

### Data

**Fig 1:**
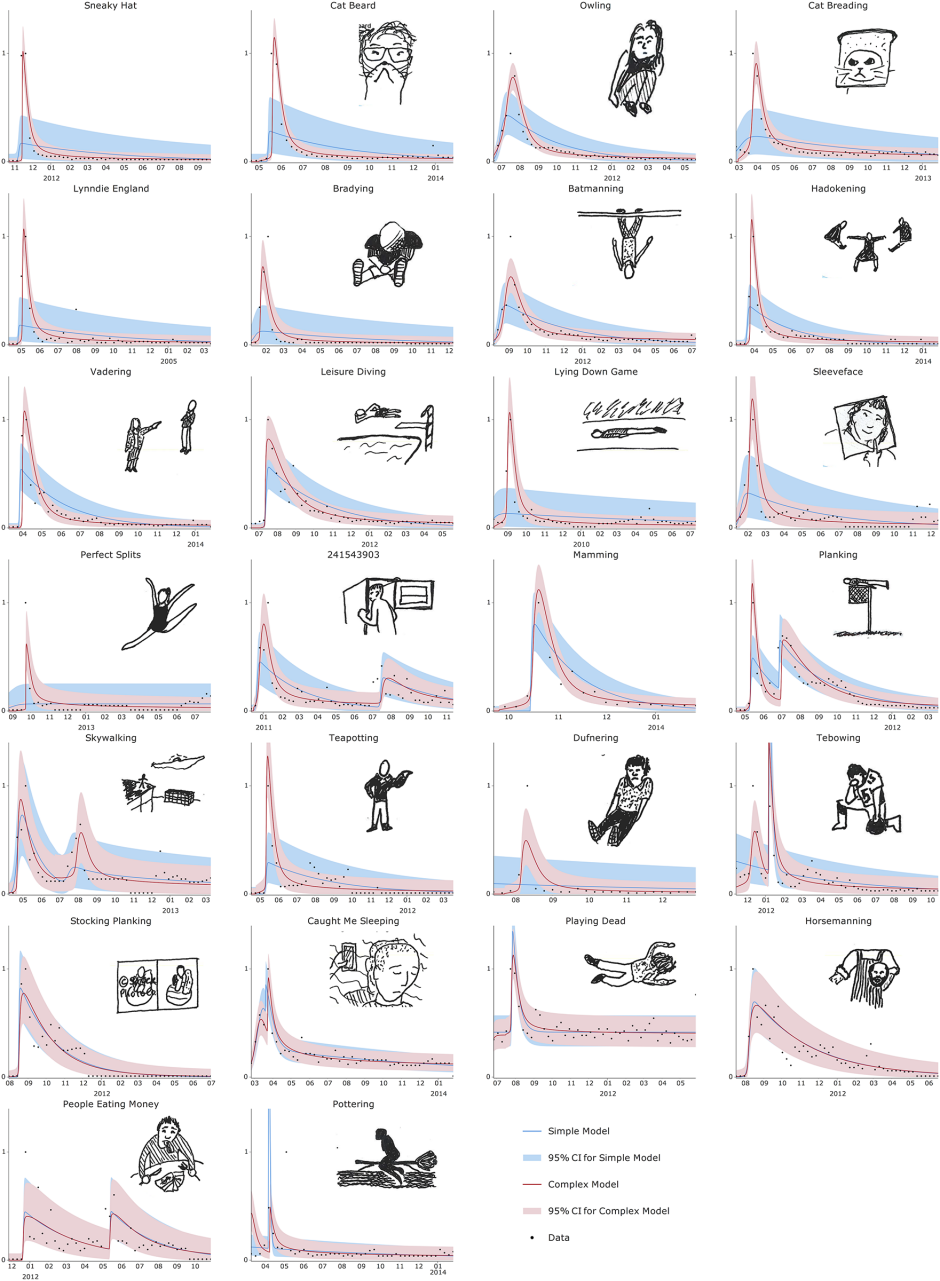
Fitted models against data for different fads. Fitted simple and complex contagion models and data for search volumes as a percentage of peak, ordered by log-likelihood difference from best fit to worst. Fads with potentially offensive content are included for completeness, but without sketches.

Our main data source was Google search volumes for a particular category of Internet meme: photo fads. These fads consist of participants uploading photos of themselves in a particular pose; descriptions of the fads are given in Table 1 and they are visualised in Fig 1.

**Table 1:** Fad descriptions. Explanations of the nomination and photo fads (excluding those that are potentially offensive).

**Nomination Fad**	**Description**
Neknomination	Consuming an alcoholic drink in one gulp.
Icebucket Challenge	Pouring a bucket of iced water over the participant’s head and / or donating to ALS research.
**Photo Fad**	**Description**
Cat Beard	Posing so that a cat’s face looks like the participant’s beard.
Owling	Crouching like an owl.
Cat Breading	Putting bread around a cat’s face.
Bradying	Copying a widely publicised photograph of NFL player Tom Brady.
Vadering	Recreating the *Star Wars* scenes in which the character Darth Vader chokes an adversary.
Hadokening	Recreating the ‘Hadoken’ move from the video game *Street Fighter*.
Batmanning	Hanging upside-down like a bat.
Lying Down Game	Lying rigidly in a public place.
Leisure Diving	Appearing to carry out a leisure activity while diving into a swimming pool.
Sleeveface	Holding a record sleeve that appears to replace the participant’s face.
241543903	Placing the participant’s head in a freezer.
Perfect Splits	Performing the splits.
Pottering	Appearing to fly on a broomstick as in *Harry Potter*.
Planking	Lying rigidly across a raised object.
Skywalking	Posing on top of a high building.
Tebowing	Copying the celebration stance of NFL player Tim Tebow.
Teapotting	Holding the participant’s arms like a teapot.
Dufnering	Copying a widely publicised photograph of golfer Jason Dufner.
Stocking Planking	Recreating a stock photograph.
Caught Me Sleeping	The participant pretends to be asleep while they are demonstrably photographing themselves.
People Eating Money	Pretenting to eat banknotes.
Playing Dead	Simulating a murder scene.
Horsemanning	Pretending to be a headless ghost.

Photo fads were chosen because they tended to have distinctive names, allowing them to be clearly identified in search data; they involved real-world behaviours that were spread by and reported on the Internet; and they were undertaken for no ostensive reason beyond their online popularity. These photo fads tended to be global phenomena, and hence took place in a population large enough to satisfy the assumptions of the ODE model.

To acquire these data, we visited the site trends.google.com and entered the relevant search term (e.g. ‘Vadering’) in the ‘Explore topics’ box, then downloaded the ‘Interest over time’ data in CSV format using the site’s download link.

We avoided selection bias by taking all 37 Photo Fads listed on the website KnowYourMeme.com (a comprehensive source of information on internet memes). The search data was obtained from Google Trends, and consisted of search volumes quoted in terms of a percentage of the peak value, and aggregated weekly. We fitted models to the 26 fads that had sufficient (greater than 15) non-zero data points to allow the dynamics of behavioural contagion to be identifiable.

### Statistical methodology

The data take the form of a set of real-valued Google Trends at discrete time points $y≔{\left(y_{t}\right)}_{t=1}^{T}$. Search data was assumed to be a proxy for the number of people taking part in the trend: infected individuals search for information about these fads at a constant rate. The noise in the data was therefore modelled as arising from overdispersed sampling with mean *μ*(*t*) ≔ *I*(*t*) + *J*(*t*), where *I*(*t*) and *J*(*t*) are solutions to the ODE fad model defined by Eq (4). For known count data the Negative Binomial distribution would be appropriate to model this overdispersed sampling, but the data provided by Google Trends is instead given as a percentage of the peak and is therefore real-valued. As such we use the Gamma distribution, which approximates the Negative Binomial in the limit of large population size and is defined on the positive real numbers, to model the noise around the mean. This gives the following likelihood function:

$$\begin{array}{c} L(y|\theta )=\prod_{t=1}^{T}\;\text{Gamma}\left(y_{t}|A\mu \left(t+\Delta t\right),r\right), \end{array}$$ (8)

where we use the ‘mean-shape’ parameterisation of the Gamma distribution. This likelihood contains three additional ‘nuisance’ parameters: *A* is the relative amplitude term to adjust for the fact that Google Trends data is quoted in terms of the fraction of the peak (given this parameter, we will make the rescaling *N* = 1 in the ODE models to remove a source of unidentifiability)—a larger *A* corresponds to a smaller imputed fad compared to the data; Δ*t* is an additive time shift to match model time with real time—a larger Δ*t* moves the fad curve left; and *r* is the Gamma shape parameter—if this is larger there is less noise in the fad at a given mean. Together with the initial conditions and constants needed to solve the ODE Systems (4) and (5) this gives parameter sets

$$\begin{array}{c} \begin{array}{ccc} \text{Complex}\;\text{contagion}\;\text{model:}\;\theta & = & (A,r,\Delta t,I\left(0\right),\beta ,\tau_{i},\tau_{j},\tau_{r},\epsilon )\text{,}\\ \text{Simple}\;\text{contagion}\;\text{model:}\;\theta & = & (A,r,\Delta t,I\left(0\right),\beta_{i},\beta_{j},\gamma ,\epsilon )\text{.} \end{array} \end{array}$$ (9)

To fit the model, *L* was maximized numerically with respect to all parameters listed above—the parameter *C* for the complex contagion model was fixed at 10 since analysis of the model structure proposed (confirmed by our numerical work) suggests that this will not be identifiable from data [19]. Integer parameters (the *τ*’s) were optimised using exhaustive (grid) methods, however our parameter spaces are too high-dimensional for this to be appropriate for all parameters—nevertheless, we were able to obtain robust maximum likelihood estimates through the use of Powell’s method [26].

For each set of fad data we calculated the Akaike Information Criterion (AIC) [27] defined as

$$\begin{array}{c} AIC=2k-2\text{ln}L^{*}\text{,} \end{array}$$ (10)

where *k* is the number of parameters for each model and L* is the maximum value of the likelihood. In this way AIC represents a trade-off between goodness of model fit and model complexity, so more complex models are not automatically selected simply because they fit the data better. There are 8 parameters in our simple contagion model and 9 parameters in our complex contagion model, meaning that these are not massively different in complexity. To quantify the level of preference for one model over another, we classified the difference in AIC between the two models into different grades of evidence, based on the suggestions of Stylianou et al. [28].

Some fads showed two clear peaks in the data. For each time series with more than one mode, we therefore fitted a model in which two separate sub-populations become infected, with the total infected fraction being the sum of infected in the sub-populations. The parameters for each population were fitted independently, except for the thresholds in the complex contagion model that were assumed constant. The AIC was again used to select between one-population and two-population versions of both contagion mechanisms.

### Prediction

**Fig 2:**
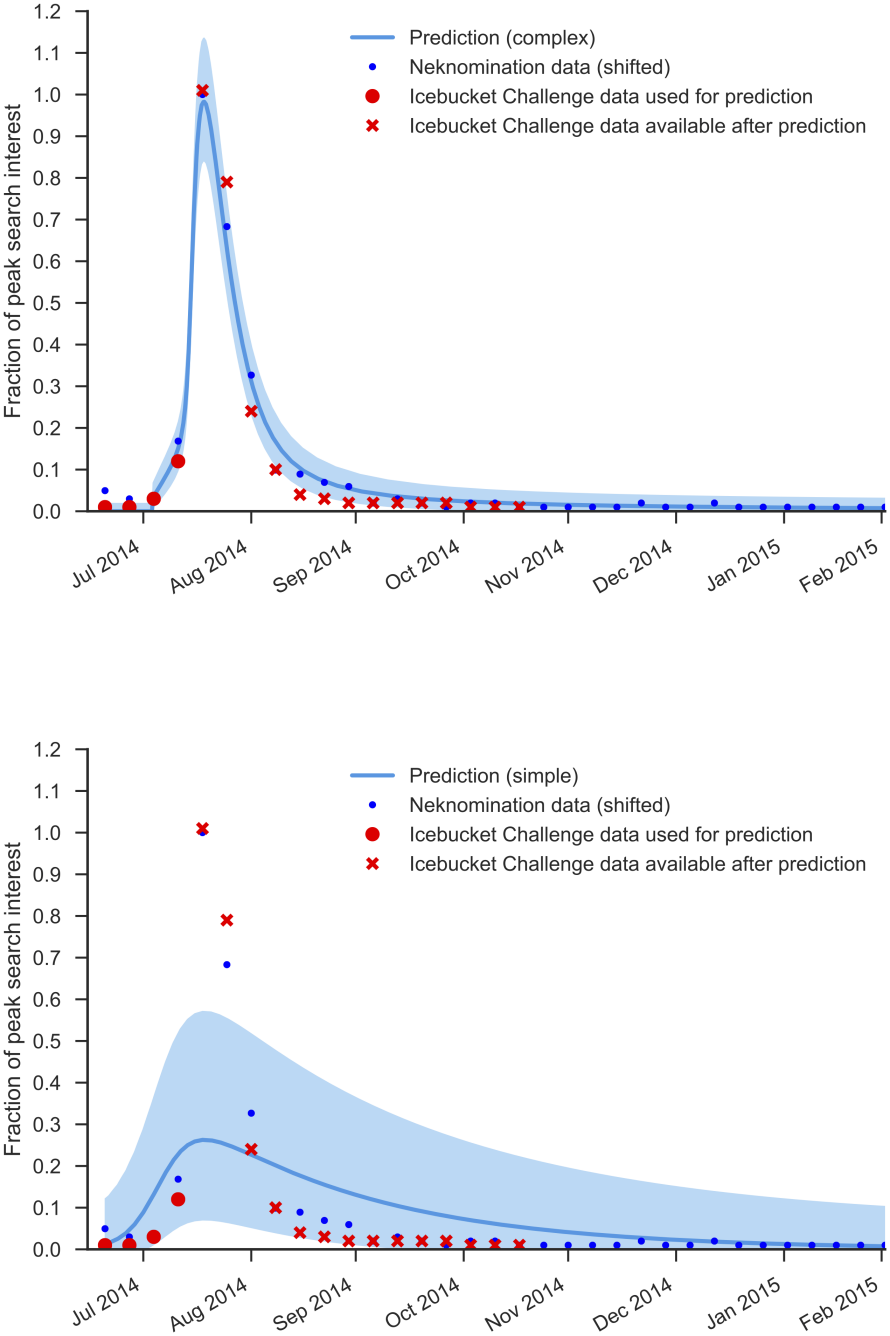
Results for the Icebucket Challenge. Prediction of search volume for Icebucket Challenge, based on data available at the time (circles) and compared to the final volume (crosses). Top plot: complex contagion model; bottom plot: simple contagion model.

The complex contagion model was used to predict the future spread of another fad, ‘ALS Icebucket Challenge’. This was a charity campaign that spread in a viral manner, with friends directly nominating each other to take part. A previous fad, ‘Neknomination’, had spread in a similar way, and so we used the parameters fitted from that fad to predict the future spread of ‘ALS Icebucket Challenge’. We made a verifiable prediction at the start of the campaign, shown in Fig 2, and overlaid the final data when the campaign had finished. The original Fig 2, unedited, is stored at https://www.facebook.com/photo.php?fbid=10100902252555809&l=931e0d22a5. The data are generally within the 95% prediction interval of the model, and the time and duration of interest in the campaign were predicted well: the peak occurred in the week predicted by the model, and the campaign was popular for the same length of time as the model.

## Results and discussion

Of these fads, 22 of 26 showed significant evidence that complex contagion was a better model for the data than simple contagion. The fitted timeseries for all fads are provided in Fig 1, ordered by log-likelihood difference. Most fads showed similar characteristics: a fast uptake, a drop in interest after the peak that was almost as fast, and then a long tail of activity taking a long time to die out.

The complex contagion model’s threshold for social influence allows it to capture the fast increase in popularity seen in most of the trends. The linear force of influence in the simple contagion model, however, means that it is slower to build to peak popularity. After the peak, the simple contagion model has a constant rate for individuals leaving the fad, leading to exponential decay in popularity. The complex contagion initially shows a fast drop in popularity as individuals see that their contacts are already taking part in the fad, but once most of the population has stopped taking part the few individuals remaining take longer to give it up. This correctly captures the ‘long tail’ of popularity seen in the data.

For a minority of fads, the simple contagion model was also adequate, but this was typically linked to few datapoints and / or poor signal quality. In terms of values for the parameters, these were quite variable between fads, which would be expected given e.g. the differing levels of effort needed to participate in each fad. Full fitted parameter values are available in S1 and S2 Files.

Table 2 shows the log-likelihood difference, $\Delta L=\text{ln}L_{c}^{*}-\text{ln}L_{s}^{*}$, between the complex contagion and the simple contagion models (the difference in number of parameters is constant for the single population models and for the double population models) and the AIC evidence grade for each fad. For 22 out of 26 fads the complex contagion model is significantly better than simple contagion. The three fads with no positive evidence for either model were noisier and had higher background search volumes than the other fads. The names of these fads (‘caught me sleeping’, ‘people eating money’, ‘playing dead’) are phrases that could appear in searches unrelated to photo fads, leading to higher noise. It is interesting that the one case where simple contagion was a significantly better model, ‘horsemanning’, was the only one started by the Internet news site *BuzzFeed* in an attempt to create a fad artificially. This suggests that a strong external driver not included in the model, such as mass media influence, can have a significant effect on the spread of a fad.

**Table 2:** Model evidence. The log-likelihood difference between the simple and complex contagion models. (***) is very strong evidence, (**) is strong evidence, (*) is positive evidence, (.) is no significant evidence for either model, (–) is strong evidence against. † means that AIC selected models with two peaks.

Photo Fad	Log-likelihood difference	AIC Evidence	
Sneaky Hat	47.4	***	
Cat Beard	44.0	***	
Owling	39.3	***	
Cat Breading	38.9	***	
Lynndie England	27.3	***	
Bradying	26.6	***	
Vadering	24.6	***	
Hadokening	24.2	***	
Batmanning	24.2	***	
Lying Down Game	17.8	***	
Leisure Diving	16.8	***	
Sleeveface	12.1	***	
241543903	11.7	***	†
Perfect Splits	11.7	***	
Mamming	10.2	***	
Pottering	9.5	***	
Planking	8.5	***	†
Skywalking	7.1	***	†
Tebowing	6.9	***	†
Teapotting	6.0	***	
Dufnering	4.7	**	
Stocking Planking	2.0	*	
Caught Me Sleeping	-0.0	.	†
People Eating Money	-1.7	.	†
Playing Dead	-1.9	.	†
Horsemanning	-3.2	–	

## Conclusions

Social influence, or the effect of others’ behaviour on our own, is important in understanding many aspects of human behaviour. Although several mechanisms have been proposed to model this influence, it has not so far been possible to distinguish between these mechanisms in observational data. Here we have shown that the observed spread of real-world behaviours linked to online trends can be explained using a complex contagion model, and demonstrate that this model provides a predictive modelling framework for real-world behaviours spread online.

## Supporting information

S1 File Complex contagion parameters. Fitted parameter values for complex contagion models. The second sets of parameters, if present, are for two-peak fits. Plain text comma-separated values.
(CSV)

S2 File Simple contagion parameters. Fitted parameter values for simple contagion models. The second sets of parameters, if present, are for two-peak fits. Plain text comma-separated values.
(CSV)
